# Naphthalene-1,8-di­amine–2-(pyrimidin-2-yl)-1*H*-perimidine (2/1)

**DOI:** 10.1107/S1600536813016796

**Published:** 2013-06-22

**Authors:** Maria Elena Cucciolito, Barbara Panunzi, Francesco Ruffo, Angela Tuzi

**Affiliations:** a Dipartimento di Scienze Chimiche, Università degli Studi di Napoli ’Federico II’, Complesso di Monte S. Angelo, Via Cinthia, 80126 Napoli, Italy; b Dipartimento di Agraria, Università degli Studi di Napoli ’Federico II’, Via Università 100, 80055 Portici, Italy

## Abstract

In the title adduct, C_15_H_10_N_4_·2C_10_H_10_N_2_, the pyrimidine ring is nearly co-planar with the heteroatomic perimidine ring, as indicated dihedral angle between their mean planes of 3.21 (11)°. The di­aminona­phthalene mol­ecules are slightly twisted [dihedral angles = 4.2 (2) and 3.0 (2)°] because of the steric encumbrance of NH_2_ groups. The perimidine and di­aminona­phthalene mol­ecules are linked by N—H⋯N hydrogen bonds, forming an *R* ^4^_4_(12) graph-set motif across an inversion center. In the crystal, alternating layers of the perimidine and di­aminona­phthalene mol­ecules are formed along [100]. In the perimidine layer, mol­ecules are π–π stacked along the *c-*axis direction with an inter­plane separation of approximately 3.4 Å.

## Related literature

For the coordination properties of perimidines, see: Morita *et al.* (2003 ▶); Cucciolito *et al.* (2013 ▶). For structural data on perimidines, see: Foces-Foces *et al.* (1993 ▶); Llamas-Saiz *et al.* (1995 ▶); Filatova *et al.* (2000 ▶); Murata *et al.* (2006 ▶); Smellie *et al.* (2011 ▶). For structural data on naphthalene-1,8-di­amine, see: Llamas-Saiz *et al.* (1991 ▶); Basaran *et al.* (1993 ▶); Batsanov *et al.* (2001 ▶). For N-rich aromatic heterocycles in organic electronics and photonics, see: Goswami *et al.* (2010 ▶); Carella *et al.* (2012 ▶); Centore *et al.* (2012 ▶). For a general survey of hydrogen bonding in crystals, see: Desiraju & Steiner (1999 ▶); Steiner (2002 ▶). For hydrogen-bonding patterns in nitro­gen-containing heterocycles, see: Centore *et al.* (2013*a* ▶,*b* ▶).
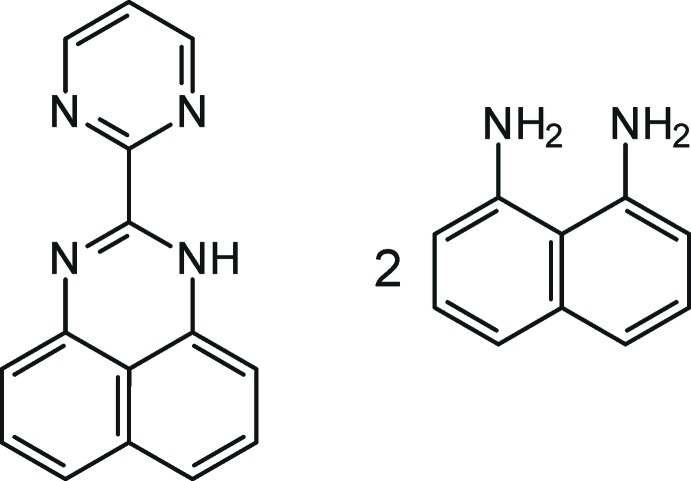


## Experimental

### 

#### Crystal data

C_15_H_10_N_4_·2C_10_H_10_N_2_
*M* *_r_* = 562.67
Monoclinic, 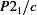

*a* = 17.083 (2) Å
*b* = 12.139 (3) Å
*c* = 13.597 (2) Å
β = 90.76 (1)°
*V* = 2819.4 (9) Å^3^
*Z* = 4
Mo *K*α radiation
μ = 0.08 mm^−1^
*T* = 173 K
0.50 × 0.45 × 0.10 mm

#### Data collection

Bruker–Nonius KappaCCD diffractometer
Absorption correction: multi-scan (*SADABS*; Bruker, 2001 ▶) *T*_min_ = 0.960, *T*_max_ = 0.992
22722 measured reflections
5184 independent reflections
2799 reflections with *I* > 2σ(*I*)
*R*_int_ = 0.080

#### Refinement

*R*[*F* ^2^ > 2σ(*F* ^2^)] = 0.065
*wR*(*F* ^2^) = 0.142
*S* = 1.06
5184 reflections
415 parameters
2 restraints
H atoms treated by a mixture of independent and constrained refinement
Δρ_max_ = 0.19 e Å^−3^
Δρ_min_ = −0.18 e Å^−3^

### 

Data collection: *COLLECT* (Nonius, 1999 ▶); cell refinement: *DIRAX/LSQ* (Duisenberg *et al.*, 2000 ▶); data reduction: *EVALCCD* (Duisenberg *et al.*, 2003 ▶); program(s) used to solve structure: *SIR97* (Altomare *et al.*, 1999 ▶); program(s) used to refine structure: *SHELXL97* (Sheldrick, 2008 ▶); molecular graphics: *ORTEP-3 for Windows* (Farrugia, 2012 ▶) and *Mercury* (Macrae *et al.*, 2006 ▶); software used to prepare material for publication: *WinGX* (Farrugia, 2012 ▶).

## Supplementary Material

Crystal structure: contains datablock(s) global, I. DOI: 10.1107/S1600536813016796/go2093sup1.cif

Structure factors: contains datablock(s) I. DOI: 10.1107/S1600536813016796/go2093Isup2.hkl

Supplementary material file. DOI: 10.1107/S1600536813016796/go2093Isup3.cml

Additional supplementary materials:  crystallographic information; 3D view; checkCIF report

## Figures and Tables

**Table 1:** Hydrogen-bond geometry (Å, °)

*D*—H⋯*A*	*D*—H	H⋯*A*	*D*⋯*A*	*D*—H⋯*A*
N1—H1⋯N2*A* ^i^	0.86 (3)	2.30 (3)	3.077 (4)	151 (2)
N1*A*—H1*C*⋯N2^ii^	0.87 (3)	2.42 (3)	3.153 (4)	142 (3)
N2*A*—H2*C*⋯N2	0.92 (3)	2.27 (3)	3.092 (4)	149 (3)
N2*A*—H2*D*⋯N1*A*	0.87 (3)	2.27 (3)	2.753 (5)	115 (2)
N1*B*—H1*F*⋯N2*B*	0.98 (4)	2.12 (3)	2.710 (5)	117 (2)

Symmetry codes: (i) 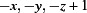; (ii) 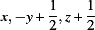.

**Fig. 1.**
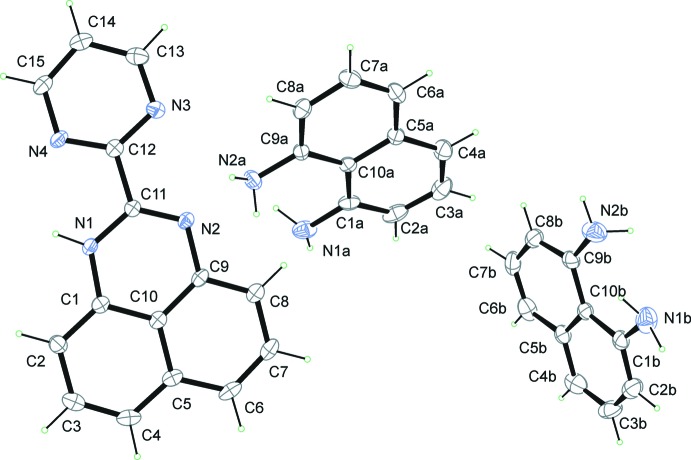
ORTEP view of the title compound. Thermal ellipsoids are drawn at 30% probability level.

**Fig. 2.**
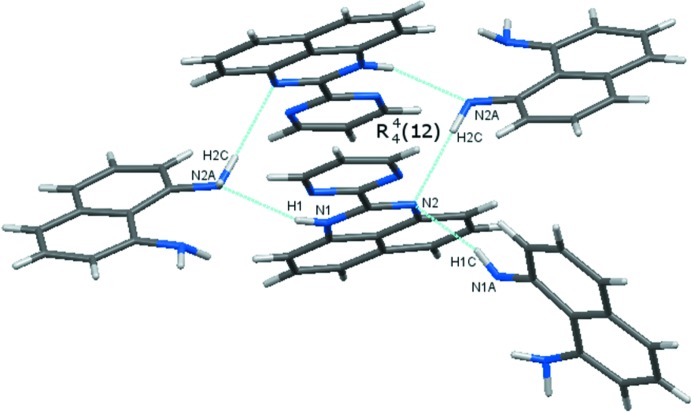
H-bonding pattern involving pyrimidinylperimidine (PIM) and naphthalene-1,8-diamine (DAN-A) molecules.

**Fig. 3.**
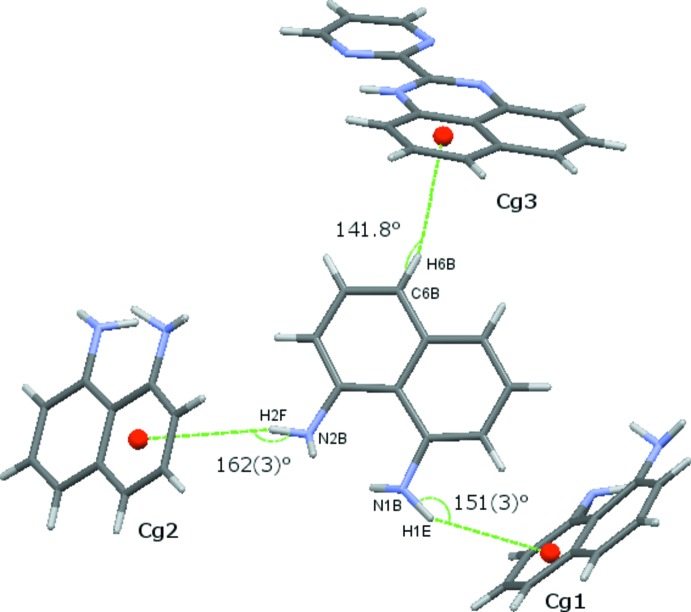
naphthalene-1,8-diamine (DAN-B) molecule involved in weak CH···π and NH···π interactions. Cg1: centroid(C5A—C10A)[x, 1 + y, z]; Cg2: centroid(C1B—C5B,C10B)[1 - x, -1/2 + y, 3/2 - z]; Cg3: centroid(C1—C5,C10)[-x, 1/2 + y, 3/2 - z].

**Fig. 4.**
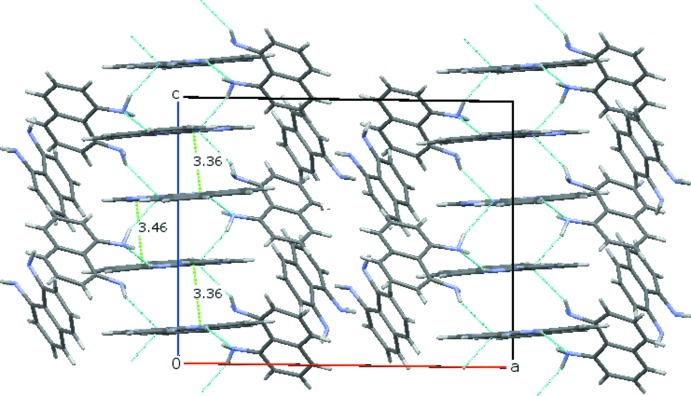
Crystal packing viewed along b axis showing short distances in the π-π stacked PIM molecules. Hydrogen bonds involving PIM molecules are drawn as dashed lines.

## supplementary crystallographic information

### Comment

During the studies on the coordination ability of perimidines containing four donor atoms (Cucciolito *et al.*, 2013) we obtained the neutral 2-(pyrimidin-2-yl)-1*H*-perimidine-naphthalene-1,8-diamine (1/2) cocrystal. In view of its N-rich aromatic character, pyrimidinylperimidine can have interest also in organic electronics and photonics (Carella *et al.*, 2012; Centore *et al.*, 2012; Murata *et al.*, 2006; Goswami *et al.*, 2010).

Few structural data are found in the literature on neutral perimidines having one amino and one imino N atom at the heterocyclic mojety (Filatova *et al.*, 2000; Foces-Foces *et al.*, 1993; Llamas-Saiz *et al.*, 1995; Murata *et al.*, 2006; Smellie *et al.*, 2011). Moreover, very few structural data on the naphthalene-1,8-diamine (DAN) ring system are found in the literature (Batsanov *et al.*, 2001; Llamas-Saiz *et al.*, 1991; Basaran *et al.*, 1993). In particular, the title compound is the second example of a perimidine bearing a pyrimidine ring at C11 with the two N atoms in *ortho* position (Morita *et al.*, 2003). In the title compound, one 2-(pyrimidin-2-yl)-1*H*-perimidine (PIM) molecule and two naphthalene-1,8-diamine (DAN-A and DAN-B) molecules are contained in the asymmetric unit (Fig.1). The aminic nature of N1 in PIM is clearly proven by location of the NH hydrogen atom in difference Fourier maps. The geometry at N1 is planar, as demonstrated by the free refinement of the attached hydrogen atom. The pyrimidine ring is nearly coplanar with the perimidine ring with a dihedral angle between their mean planes of 3.21 (11)°. In DAN-A and DAN-B molecules the geometric parameters are very similar to literature data of pure DAN (Llamas-Saiz *et al.*, 1991; Basaran *et al.*,1993) and of octafluoronaphthalene-naphthalene-1,8-diamine co-crystal (Batsanov *et al.*, 2001). As usually found, the molecule adopts a slightly twisted conformation due to the steric encumbrance of the two adjacent NH_2_ groups. The hydrogen atoms of NH_2_ groups, located in difference Fourier maps, confirm that two different orientations are adopted by the amine groups, with inward hydrogen atoms forced into intramolecular contacts. It is not clear if such kind of contacts, associated to a steric strain of the DAN molecule, can be considered as weak intermolecular hydrogen bonds or as constrain-due interactions. However, the refined positions of NH_2_ hydrogen atoms give a N–H···N geometry that falls in the range of weak hydrogen bonds [D(*X*···A) = 3.0–4.0 Å and θ(*X*–A···H) = 90–180° (Desiraju & Steiner, 1999)].

PIM and DAN-A molecules are involved in N–H···N hydrogen bonds (Fig.2) forming the *R*^4^_4_(12) graph-set motif across an inversion center. The amine N1 atom acts as donor towards N2A^i^ (i = -*x*,-*y*,1 - *z*) and imine N2 atom acts as bifurcated acceptor from N1A and from N2A^ii^ (ii = *x*, 1/2 - *y*, *z* - 1/2).

The DAN-B molecules are not involved in classic intermolecular hydrogen bonds, but only in weak CH···π and NH···π interactions (Fig.3) (N1B—H1E··· *Cg*1(C5A—C10A)[*x*, 1 + *y*, *z*] = 0.98 (4), 3.15 (4) Å, 151 (3)°; N2B—H2F···*Cg*2(C1B—C5B,C10B)[1 - *x*, -1/2 + *y*, 3/2 - *z*] = 0.91 (4), 3.35 (4) Å, 162 (3)°;C6B—H6B···*Cg*3(C1—C5,C10)[-*x*, 1/2 + *y*,3/2 - *z*] = 0.95, 3.632 Å, 141.8°).

In the crystal packing (Fig. 4) layers of PIM molecules are alternate to double layers of DAN-A and DAN-B molecules along [1 0 0] direction. In the perimidine layer molecules are π···π stacked along *c* with interplanar separation of approximately 3.4 Å.

### Experimental

2-(pyrimidin-2-yl)-1*H*-perimidine was obtained from naphthalene-1,8-diamine and pyrimidine-2-carbonitrile, details on the synthesis will be reported in a forthcoming work. Block-shaped crystals of title compound were obtained by evaporation of an ethyl acetate/cyclohexane solution containing 2-(pyrimidin-2-yl)-1*H*-perimidine and naphthalene-1,8-diamine.

### Refinement

The NH hydrogen atoms were located in difference Fourier maps and refined with *U*_iso_=1.2*U*_eq_(N) of the carrier atom. All other H atoms were generated stereochemically and refined by the riding model with C–H=0.95 Å and *U*_iso_(H)=1.2*U*_eq_(C). Anti-bumping restraints were used in the last stage of the refinement.

### Crystal data

**Table:**

C_15_H_10_N_4_·2C_10_H_10_N_2_	*F*(000) = 1184
*M**_r_* = 562.67	*D*_x_ = 1.326 Mg m^−^^3^
Monoclinic, *P*2_1_/*c*	Mo *K*α radiation, λ = 0.71073 Å
Hall symbol: -P 2ybc	Cell parameters from 126 reflections
*a* = 17.083 (2) Å	θ = 3.4–21.8°
*b* = 12.139 (3) Å	µ = 0.08 mm^−^^1^
*c* = 13.597 (2) Å	*T* = 173 K
β = 90.76 (1)°	Block, orange
*V* = 2819.4 (9) Å^3^	0.50 × 0.45 × 0.10 mm
*Z* = 4	

### Data collection

**Table:**

Bruker–Nonius KappaCCD diffractometer	5184 independent reflections
Radiation source: normal-focus sealed tube	2799 reflections with *I* > 2σ(*I*)
Graphite monochromator	*R*_int_ = 0.080
Detector resolution: 9 pixels mm^-1^	θ_max_ = 25.4°, θ_min_ = 3.2°
CCD rotation images, thick slices scans	*h* = −20→20
Absorption correction: multi-scan (*SADABS*; Bruker, 2001)	*k* = −14→14
*T*_min_ = 0.960, *T*_max_ = 0.992	*l* = −16→16
22722 measured reflections	

### Refinement

**Table:**

Refinement on *F*^2^	Primary atom site location: structure-invariant direct methods
Least-squares matrix: full	Secondary atom site location: difference Fourier map
*R*[*F*^2^ > 2σ(*F*^2^)] = 0.065	Hydrogen site location: inferred from neighbouring sites
*wR*(*F*^2^) = 0.142	H atoms treated by a mixture of independent and constrained refinement
*S* = 1.06	*w* = 1/[σ^2^(*F*_o_^2^) + (0.0402*P*)^2^ + 1.5806*P*] where *P* = (*F*_o_^2^ + 2*F*_c_^2^)/3
5184 reflections	(Δ/σ)_max_ < 0.001
415 parameters	Δρ_max_ = 0.19 e Å^−^^3^
2 restraints	Δρ_min_ = −0.18 e Å^−^^3^

### Special details

**Table:**

Geometry. All e.s.d.'s (except the e.s.d. in the dihedral angle between two l.s. planes) are estimated using the full covariance matrix. The cell e.s.d.'s are taken into account individually in the estimation of e.s.d.'s in distances, angles and torsion angles; correlations between e.s.d.'s in cell parameters are only used when they are defined by crystal symmetry. An approximate (isotropic) treatment of cell e.s.d.'s is used for estimating e.s.d.'s involving l.s. planes.
Refinement. Refinement of *F*^2^ against ALL reflections. The weighted *R*-factor *wR* and goodness of fit *S* are based on *F*^2^, conventional *R*-factors *R* are based on *F*, with *F* set to zero for negative *F*^2^. The threshold expression of *F*^2^ > σ(*F*^2^) is used only for calculating *R*-factors(gt) *etc*. and is not relevant to the choice of reflections for refinement. *R*-factors based on *F*^2^ are statistically about twice as large as those based on *F*, and *R*- factors based on ALL data will be even larger.

### Fractional atomic coordinates and isotropic or equivalent isotropic displacement parameters (Å^2^)

**Table:**

	*x*	*y*	*z*	*U*_iso_*/*U*_eq_	
N1	−0.06299 (14)	0.0273 (2)	0.36212 (17)	0.0276 (6)	
H1	−0.0848 (16)	−0.037 (2)	0.365 (2)	0.033*	
N2	0.05732 (13)	0.12080 (19)	0.37659 (16)	0.0269 (6)	
N3	0.13410 (14)	−0.0767 (2)	0.38241 (18)	0.0352 (6)	
N4	0.00939 (14)	−0.16518 (19)	0.37372 (17)	0.0309 (6)	
N1A	0.17020 (18)	0.3184 (3)	0.7032 (2)	0.0500 (9)	
H1C	0.1574 (19)	0.358 (3)	0.753 (3)	0.060*	
H1D	0.1615 (19)	0.244 (3)	0.7159 (19)	0.060*	
N2A	0.16490 (15)	0.1606 (2)	0.5578 (2)	0.0400 (7)	
H2C	0.1462 (18)	0.126 (3)	0.502 (2)	0.048*	
H2D	0.1347 (18)	0.216 (3)	0.5700 (19)	0.048*	
N1B	0.44606 (19)	0.9546 (3)	0.6366 (2)	0.0624 (10)	
H1E	0.430 (2)	1.011 (3)	0.588 (3)	0.075*	
H1F	0.4405 (19)	0.878 (3)	0.615 (2)	0.075*	
N2B	0.48654 (19)	0.7540 (3)	0.7084 (3)	0.0634 (10)	
H2E	0.5269 (19)	0.805 (3)	0.702 (3)	0.076*	
H2F	0.509 (2)	0.686 (3)	0.709 (3)	0.076*	
C1	−0.10821 (16)	0.1228 (2)	0.3582 (2)	0.0270 (7)	
C2	−0.18822 (16)	0.1208 (3)	0.3468 (2)	0.0334 (7)	
H2	−0.2158	0.0532	0.3403	0.040*	
C3	−0.22799 (18)	0.2222 (3)	0.3449 (2)	0.0407 (8)	
H3	−0.2833	0.2224	0.3367	0.049*	
C4	−0.18985 (19)	0.3200 (3)	0.3547 (2)	0.0391 (8)	
H4	−0.2190	0.3867	0.3543	0.047*	
C5	−0.10814 (17)	0.3240 (2)	0.3652 (2)	0.0299 (7)	
C6	−0.0637 (2)	0.4223 (2)	0.3748 (2)	0.0380 (8)	
H6	−0.0895	0.4916	0.3748	0.046*	
C7	0.0159 (2)	0.4181 (2)	0.3840 (2)	0.0392 (8)	
H7	0.0444	0.4849	0.3906	0.047*	
C8	0.05660 (18)	0.3185 (2)	0.3841 (2)	0.0343 (8)	
H8	0.1121	0.3181	0.3898	0.041*	
C9	0.01606 (17)	0.2209 (2)	0.3757 (2)	0.0266 (7)	
C10	−0.06663 (16)	0.2226 (2)	0.36614 (19)	0.0256 (7)	
C11	0.01603 (16)	0.0311 (2)	0.3711 (2)	0.0244 (7)	
C12	0.05592 (16)	−0.0776 (2)	0.37559 (19)	0.0253 (7)	
C13	0.16700 (19)	−0.1763 (3)	0.3871 (2)	0.0416 (8)	
H13	0.2225	−0.1807	0.3915	0.050*	
C14	0.12521 (19)	−0.2720 (3)	0.3860 (2)	0.0366 (8)	
H14	0.1502	−0.3418	0.3899	0.044*	
C15	0.04540 (19)	−0.2627 (2)	0.3789 (2)	0.0347 (8)	
H15	0.0146	−0.3278	0.3776	0.042*	
C1A	0.24650 (19)	0.3402 (3)	0.6713 (2)	0.0370 (8)	
C2A	0.2866 (2)	0.4285 (3)	0.7099 (2)	0.0491 (10)	
H2A	0.2626	0.4724	0.7588	0.059*	
C3A	0.3619 (2)	0.4554 (3)	0.6791 (3)	0.0533 (10)	
H3A	0.3874	0.5183	0.7058	0.064*	
C4A	0.39903 (19)	0.3927 (3)	0.6114 (3)	0.0454 (9)	
H4A	0.4507	0.4110	0.5921	0.054*	
C5A	0.36083 (17)	0.3002 (2)	0.5698 (2)	0.0320 (7)	
C6A	0.40047 (18)	0.2335 (3)	0.5023 (2)	0.0395 (8)	
H6A	0.4532	0.2496	0.4864	0.047*	
C7A	0.36393 (19)	0.1463 (3)	0.4595 (2)	0.0419 (9)	
H7A	0.3919	0.1002	0.4157	0.050*	
C8A	0.28593 (18)	0.1239 (3)	0.4792 (2)	0.0374 (8)	
H8A	0.2610	0.0635	0.4472	0.045*	
C9A	0.24415 (16)	0.1870 (2)	0.5438 (2)	0.0293 (7)	
C10A	0.28182 (16)	0.2759 (2)	0.5957 (2)	0.0285 (7)	
C1B	0.40270 (18)	0.9666 (3)	0.7235 (3)	0.0426 (9)	
C2B	0.3652 (2)	1.0638 (3)	0.7392 (3)	0.0549 (10)	
H2B	0.3690	1.1203	0.6912	0.066*	
C3B	0.3216 (2)	1.0833 (3)	0.8226 (3)	0.0632 (12)	
H3B	0.2972	1.1529	0.8315	0.076*	
C4B	0.3137 (2)	1.0033 (3)	0.8918 (3)	0.0563 (11)	
H4B	0.2829	1.0162	0.9482	0.068*	
C5B	0.35172 (18)	0.9003 (3)	0.8793 (3)	0.0409 (9)	
C6B	0.3421 (2)	0.8162 (4)	0.9495 (3)	0.0567 (10)	
H6B	0.3122	0.8297	1.0067	0.068*	
C7B	0.3754 (2)	0.7156 (3)	0.9355 (3)	0.0601 (11)	
H7B	0.3661	0.6581	0.9813	0.072*	
C8B	0.4223 (2)	0.6962 (3)	0.8556 (3)	0.0514 (10)	
H8B	0.4454	0.6256	0.8478	0.062*	
C9B	0.43639 (18)	0.7761 (3)	0.7875 (3)	0.0406 (8)	
C10B	0.39817 (17)	0.8811 (3)	0.7954 (2)	0.0344 (8)	

### Atomic displacement parameters (Å^2^)

**Table:**

	*U*^11^	*U*^22^	*U*^33^	*U*^12^	*U*^13^	*U*^23^
N1	0.0295 (15)	0.0195 (14)	0.0338 (15)	−0.0026 (11)	0.0021 (12)	−0.0003 (12)
N2	0.0309 (14)	0.0224 (14)	0.0274 (14)	−0.0013 (11)	0.0025 (11)	0.0024 (11)
N3	0.0298 (15)	0.0333 (16)	0.0423 (17)	0.0030 (12)	−0.0031 (12)	−0.0014 (12)
N4	0.0376 (15)	0.0196 (14)	0.0356 (15)	0.0006 (12)	0.0062 (12)	0.0015 (11)
N1A	0.058 (2)	0.049 (2)	0.044 (2)	0.0175 (17)	0.0180 (16)	0.0009 (15)
N2A	0.0279 (16)	0.0462 (19)	0.0457 (18)	−0.0023 (13)	−0.0014 (13)	0.0048 (14)
N1B	0.067 (2)	0.070 (2)	0.050 (2)	−0.0128 (19)	−0.0006 (18)	0.0161 (18)
N2B	0.054 (2)	0.064 (3)	0.072 (2)	0.0110 (17)	0.0032 (19)	−0.018 (2)
C1	0.0333 (18)	0.0265 (17)	0.0213 (16)	0.0010 (14)	0.0027 (13)	0.0027 (13)
C2	0.0289 (18)	0.0361 (19)	0.0351 (19)	0.0000 (15)	−0.0007 (14)	0.0012 (15)
C3	0.0321 (19)	0.052 (2)	0.038 (2)	0.0099 (17)	0.0003 (15)	0.0036 (17)
C4	0.045 (2)	0.038 (2)	0.0336 (19)	0.0172 (17)	0.0032 (16)	0.0048 (16)
C5	0.042 (2)	0.0270 (17)	0.0202 (16)	0.0066 (15)	0.0034 (14)	−0.0010 (13)
C6	0.063 (2)	0.0218 (17)	0.0294 (19)	0.0078 (16)	0.0017 (17)	0.0026 (14)
C7	0.055 (2)	0.0240 (18)	0.038 (2)	−0.0063 (16)	−0.0025 (17)	−0.0004 (15)
C8	0.0406 (19)	0.0282 (18)	0.0340 (19)	−0.0022 (15)	−0.0011 (15)	0.0007 (14)
C9	0.0389 (18)	0.0211 (16)	0.0199 (16)	−0.0015 (14)	0.0016 (14)	−0.0012 (13)
C10	0.0341 (18)	0.0263 (17)	0.0166 (15)	−0.0006 (14)	0.0015 (13)	−0.0019 (12)
C11	0.0280 (17)	0.0256 (17)	0.0198 (16)	−0.0006 (14)	0.0031 (13)	−0.0004 (13)
C12	0.0331 (18)	0.0282 (17)	0.0146 (16)	−0.0002 (14)	0.0028 (13)	−0.0010 (13)
C13	0.0386 (19)	0.044 (2)	0.042 (2)	0.0117 (18)	−0.0039 (16)	−0.0006 (16)
C14	0.050 (2)	0.0298 (19)	0.0299 (19)	0.0106 (16)	0.0010 (16)	0.0010 (14)
C15	0.048 (2)	0.0226 (18)	0.0336 (19)	−0.0010 (15)	0.0067 (16)	0.0005 (14)
C1A	0.045 (2)	0.036 (2)	0.0293 (19)	0.0145 (16)	−0.0003 (16)	0.0048 (15)
C2A	0.079 (3)	0.036 (2)	0.032 (2)	0.017 (2)	−0.0075 (19)	−0.0048 (17)
C3A	0.071 (3)	0.037 (2)	0.051 (2)	−0.008 (2)	−0.029 (2)	−0.0005 (19)
C4A	0.040 (2)	0.047 (2)	0.048 (2)	−0.0062 (17)	−0.0169 (17)	0.0059 (18)
C5A	0.0319 (18)	0.0334 (19)	0.0305 (18)	−0.0006 (14)	−0.0079 (14)	0.0039 (14)
C6A	0.0281 (18)	0.051 (2)	0.040 (2)	−0.0026 (16)	0.0022 (16)	0.0068 (17)
C7A	0.040 (2)	0.050 (2)	0.036 (2)	0.0082 (17)	0.0068 (16)	−0.0053 (16)
C8A	0.046 (2)	0.0325 (19)	0.0333 (19)	−0.0042 (16)	−0.0010 (16)	−0.0041 (15)
C9A	0.0284 (17)	0.0299 (17)	0.0295 (18)	−0.0015 (14)	−0.0035 (14)	0.0068 (14)
C10A	0.0296 (17)	0.0302 (17)	0.0256 (17)	0.0039 (13)	−0.0046 (14)	0.0078 (14)
C1B	0.0345 (19)	0.044 (2)	0.049 (2)	−0.0091 (17)	−0.0123 (17)	0.0052 (18)
C2B	0.059 (2)	0.041 (2)	0.065 (3)	−0.0044 (19)	−0.022 (2)	0.004 (2)
C3B	0.065 (3)	0.036 (2)	0.088 (3)	0.009 (2)	−0.028 (2)	−0.015 (2)
C4B	0.047 (2)	0.063 (3)	0.059 (3)	0.002 (2)	−0.0093 (19)	−0.027 (2)
C5B	0.0336 (19)	0.044 (2)	0.045 (2)	−0.0066 (16)	−0.0081 (17)	−0.0036 (17)
C6B	0.044 (2)	0.079 (3)	0.047 (2)	−0.011 (2)	−0.0019 (18)	0.007 (2)
C7B	0.050 (2)	0.062 (3)	0.067 (3)	−0.022 (2)	−0.017 (2)	0.031 (2)
C8B	0.045 (2)	0.038 (2)	0.071 (3)	−0.0052 (17)	−0.023 (2)	0.005 (2)
C9B	0.0308 (18)	0.044 (2)	0.047 (2)	−0.0023 (16)	−0.0101 (16)	−0.0069 (18)
C10B	0.0308 (17)	0.0348 (19)	0.038 (2)	−0.0050 (15)	−0.0076 (15)	−0.0021 (15)

### Geometric parameters (Å, º)

**Table:**

N1—C11	1.355 (3)	C13—C14	1.364 (4)
N1—C1	1.393 (3)	C13—H13	0.9500
N1—H1	0.86 (3)	C14—C15	1.370 (4)
N2—C11	1.299 (3)	C14—H14	0.9500
N2—C9	1.404 (3)	C15—H15	0.9500
N3—C13	1.334 (4)	C1A—C2A	1.373 (4)
N3—C12	1.338 (3)	C1A—C10A	1.431 (4)
N4—C12	1.328 (3)	C2A—C3A	1.396 (5)
N4—C15	1.335 (3)	C2A—H2A	0.9500
N1A—C1A	1.404 (4)	C3A—C4A	1.358 (5)
N1A—H1C	0.87 (3)	C3A—H3A	0.9500
N1A—H1D	0.93 (3)	C4A—C5A	1.413 (4)
N2A—C9A	1.407 (4)	C4A—H4A	0.9500
N2A—H2C	0.92 (3)	C5A—C6A	1.405 (4)
N2A—H2D	0.87 (3)	C5A—C10A	1.430 (4)
N1B—C1B	1.411 (4)	C6A—C7A	1.356 (4)
N1B—H1E	0.98 (4)	C6A—H6A	0.9500
N1B—H1F	0.98 (4)	C7A—C8A	1.390 (4)
N2B—C9B	1.410 (4)	C7A—H7A	0.9500
N2B—H2E	0.93 (4)	C8A—C9A	1.374 (4)
N2B—H2F	0.91 (4)	C8A—H8A	0.9500
C1—C2	1.374 (4)	C9A—C10A	1.436 (4)
C1—C10	1.408 (4)	C1B—C2B	1.361 (5)
C2—C3	1.406 (4)	C1B—C10B	1.428 (4)
C2—H2	0.9500	C2B—C3B	1.385 (5)
C3—C4	1.360 (4)	C2B—H2B	0.9500
C3—H3	0.9500	C3B—C4B	1.360 (5)
C4—C5	1.402 (4)	C3B—H3B	0.9500
C4—H4	0.9500	C4B—C5B	1.420 (5)
C5—C6	1.420 (4)	C4B—H4B	0.9500
C5—C10	1.421 (4)	C5B—C6B	1.408 (5)
C6—C7	1.364 (4)	C5B—C10B	1.417 (4)
C6—H6	0.9500	C6B—C7B	1.361 (5)
C7—C8	1.395 (4)	C6B—H6B	0.9500
C7—H7	0.9500	C7B—C8B	1.380 (5)
C8—C9	1.377 (4)	C7B—H7B	0.9500
C8—H8	0.9500	C8B—C9B	1.363 (5)
C9—C10	1.417 (4)	C8B—H8B	0.9500
C11—C12	1.486 (4)	C9B—C10B	1.437 (4)
			
C11—N1—C1	121.8 (2)	C14—C15—H15	118.8
C11—N1—H1	117.2 (19)	C2A—C1A—N1A	119.3 (3)
C1—N1—H1	120.7 (19)	C2A—C1A—C10A	119.2 (3)
C11—N2—C9	116.9 (2)	N1A—C1A—C10A	121.4 (3)
C13—N3—C12	114.6 (3)	C1A—C2A—C3A	121.6 (3)
C12—N4—C15	115.7 (3)	C1A—C2A—H2A	119.2
C1A—N1A—H1C	113 (2)	C3A—C2A—H2A	119.2
C1A—N1A—H1D	113 (2)	C4A—C3A—C2A	120.9 (3)
H1C—N1A—H1D	111 (3)	C4A—C3A—H3A	119.6
C9A—N2A—H2C	108.4 (19)	C2A—C3A—H3A	119.6
C9A—N2A—H2D	115 (2)	C3A—C4A—C5A	119.9 (3)
H2C—N2A—H2D	108 (3)	C3A—C4A—H4A	120.0
C1B—N1B—H1E	110 (2)	C5A—C4A—H4A	120.0
C1B—N1B—H1F	108 (2)	C6A—C5A—C4A	119.7 (3)
H1E—N1B—H1F	115 (3)	C6A—C5A—C10A	120.4 (3)
C9B—N2B—H2E	113 (2)	C4A—C5A—C10A	119.9 (3)
C9B—N2B—H2F	115 (2)	C7A—C6A—C5A	120.5 (3)
H2E—N2B—H2F	108 (3)	C7A—C6A—H6A	119.8
C2—C1—N1	122.7 (3)	C5A—C6A—H6A	119.8
C2—C1—C10	121.6 (3)	C6A—C7A—C8A	120.5 (3)
N1—C1—C10	115.7 (3)	C6A—C7A—H7A	119.7
C1—C2—C3	117.8 (3)	C8A—C7A—H7A	119.7
C1—C2—H2	121.1	C9A—C8A—C7A	121.4 (3)
C3—C2—H2	121.1	C9A—C8A—H8A	119.3
C4—C3—C2	122.1 (3)	C7A—C8A—H8A	119.3
C4—C3—H3	118.9	C8A—C9A—N2A	117.9 (3)
C2—C3—H3	118.9	C8A—C9A—C10A	120.0 (3)
C3—C4—C5	121.0 (3)	N2A—C9A—C10A	122.1 (3)
C3—C4—H4	119.5	C5A—C10A—C1A	118.2 (3)
C5—C4—H4	119.5	C5A—C10A—C9A	116.9 (3)
C4—C5—C6	124.7 (3)	C1A—C10A—C9A	124.9 (3)
C4—C5—C10	117.8 (3)	C2B—C1B—N1B	118.3 (3)
C6—C5—C10	117.5 (3)	C2B—C1B—C10B	119.5 (3)
C7—C6—C5	120.5 (3)	N1B—C1B—C10B	122.2 (3)
C7—C6—H6	119.7	C1B—C2B—C3B	122.4 (4)
C5—C6—H6	119.7	C1B—C2B—H2B	118.8
C6—C7—C8	121.9 (3)	C3B—C2B—H2B	118.8
C6—C7—H7	119.0	C4B—C3B—C2B	120.2 (4)
C8—C7—H7	119.0	C4B—C3B—H3B	119.9
C9—C8—C7	119.7 (3)	C2B—C3B—H3B	119.9
C9—C8—H8	120.1	C3B—C4B—C5B	119.8 (4)
C7—C8—H8	120.1	C3B—C4B—H4B	120.1
C8—C9—N2	119.5 (3)	C5B—C4B—H4B	120.1
C8—C9—C10	119.6 (3)	C6B—C5B—C10B	119.9 (3)
N2—C9—C10	120.9 (3)	C6B—C5B—C4B	120.0 (4)
C1—C10—C9	119.7 (3)	C10B—C5B—C4B	120.1 (3)
C1—C10—C5	119.6 (3)	C7B—C6B—C5B	120.2 (4)
C9—C10—C5	120.7 (3)	C7B—C6B—H6B	119.9
N2—C11—N1	125.0 (3)	C5B—C6B—H6B	119.9
N2—C11—C12	119.6 (3)	C6B—C7B—C8B	120.8 (3)
N1—C11—C12	115.4 (2)	C6B—C7B—H7B	119.6
N4—C12—N3	127.2 (3)	C8B—C7B—H7B	119.6
N4—C12—C11	115.9 (3)	C9B—C8B—C7B	121.5 (3)
N3—C12—C11	116.9 (3)	C9B—C8B—H8B	119.2
N3—C13—C14	123.5 (3)	C7B—C8B—H8B	119.2
N3—C13—H13	118.3	C8B—C9B—N2B	119.8 (3)
C14—C13—H13	118.3	C8B—C9B—C10B	119.7 (3)
C13—C14—C15	116.7 (3)	N2B—C9B—C10B	120.4 (3)
C13—C14—H14	121.6	C5B—C10B—C1B	117.9 (3)
C15—C14—H14	121.6	C5B—C10B—C9B	117.7 (3)
N4—C15—C14	122.3 (3)	C1B—C10B—C9B	124.4 (3)
N4—C15—H15	118.8		
			
C11—N1—C1—C2	178.4 (3)	C1A—C2A—C3A—C4A	−1.9 (5)
C11—N1—C1—C10	−1.3 (4)	C2A—C3A—C4A—C5A	1.3 (5)
N1—C1—C2—C3	179.3 (3)	C3A—C4A—C5A—C6A	−177.9 (3)
C10—C1—C2—C3	−1.1 (4)	C3A—C4A—C5A—C10A	2.9 (5)
C1—C2—C3—C4	−0.4 (5)	C4A—C5A—C6A—C7A	−178.1 (3)
C2—C3—C4—C5	1.2 (5)	C10A—C5A—C6A—C7A	1.1 (5)
C3—C4—C5—C6	179.3 (3)	C5A—C6A—C7A—C8A	2.3 (5)
C3—C4—C5—C10	−0.4 (4)	C6A—C7A—C8A—C9A	−1.6 (5)
C4—C5—C6—C7	−179.5 (3)	C7A—C8A—C9A—N2A	178.4 (3)
C10—C5—C6—C7	0.3 (4)	C7A—C8A—C9A—C10A	−2.4 (4)
C5—C6—C7—C8	0.3 (5)	C6A—C5A—C10A—C1A	174.4 (3)
C6—C7—C8—C9	−0.7 (5)	C4A—C5A—C10A—C1A	−6.4 (4)
C7—C8—C9—N2	−179.5 (3)	C6A—C5A—C10A—C9A	−4.9 (4)
C7—C8—C9—C10	0.7 (4)	C4A—C5A—C10A—C9A	174.3 (3)
C11—N2—C9—C8	178.3 (3)	C2A—C1A—C10A—C5A	5.8 (4)
C11—N2—C9—C10	−1.9 (4)	N1A—C1A—C10A—C5A	−177.6 (3)
C2—C1—C10—C9	−178.7 (3)	C2A—C1A—C10A—C9A	−175.0 (3)
N1—C1—C10—C9	1.0 (4)	N1A—C1A—C10A—C9A	1.6 (4)
C2—C1—C10—C5	1.8 (4)	C8A—C9A—C10A—C5A	5.5 (4)
N1—C1—C10—C5	−178.5 (2)	N2A—C9A—C10A—C5A	−175.3 (3)
C8—C9—C10—C1	−179.6 (3)	C8A—C9A—C10A—C1A	−173.7 (3)
N2—C9—C10—C1	0.5 (4)	N2A—C9A—C10A—C1A	5.4 (4)
C8—C9—C10—C5	−0.1 (4)	N1B—C1B—C2B—C3B	−179.6 (3)
N2—C9—C10—C5	−180.0 (2)	C10B—C1B—C2B—C3B	−0.6 (5)
C4—C5—C10—C1	−1.1 (4)	C1B—C2B—C3B—C4B	−1.3 (6)
C6—C5—C10—C1	179.2 (3)	C2B—C3B—C4B—C5B	1.4 (5)
C4—C5—C10—C9	179.4 (3)	C3B—C4B—C5B—C6B	−178.4 (3)
C6—C5—C10—C9	−0.3 (4)	C3B—C4B—C5B—C10B	0.4 (5)
C9—N2—C11—N1	1.8 (4)	C10B—C5B—C6B—C7B	−1.9 (5)
C9—N2—C11—C12	−177.6 (2)	C4B—C5B—C6B—C7B	176.9 (3)
C1—N1—C11—N2	−0.2 (4)	C5B—C6B—C7B—C8B	3.5 (5)
C1—N1—C11—C12	179.2 (2)	C6B—C7B—C8B—C9B	−0.7 (5)
C15—N4—C12—N3	−0.3 (4)	C7B—C8B—C9B—N2B	178.2 (3)
C15—N4—C12—C11	−179.3 (2)	C7B—C8B—C9B—C10B	−3.6 (5)
C13—N3—C12—N4	0.4 (4)	C6B—C5B—C10B—C1B	176.6 (3)
C13—N3—C12—C11	179.4 (2)	C4B—C5B—C10B—C1B	−2.2 (4)
N2—C11—C12—N4	177.2 (2)	C6B—C5B—C10B—C9B	−2.3 (4)
N1—C11—C12—N4	−2.2 (3)	C4B—C5B—C10B—C9B	178.9 (3)
N2—C11—C12—N3	−1.9 (4)	C2B—C1B—C10B—C5B	2.3 (4)
N1—C11—C12—N3	178.7 (2)	N1B—C1B—C10B—C5B	−178.7 (3)
C12—N3—C13—C14	−0.5 (4)	C2B—C1B—C10B—C9B	−178.9 (3)
N3—C13—C14—C15	0.5 (5)	N1B—C1B—C10B—C9B	0.1 (5)
C12—N4—C15—C14	0.2 (4)	C8B—C9B—C10B—C5B	5.0 (4)
C13—C14—C15—N4	−0.3 (4)	N2B—C9B—C10B—C5B	−176.8 (3)
N1A—C1A—C2A—C3A	−178.4 (3)	C8B—C9B—C10B—C1B	−173.8 (3)
C10A—C1A—C2A—C3A	−1.8 (5)	N2B—C9B—C10B—C1B	4.4 (5)

### Hydrogen-bond geometry (Å, º)

**Table:**

*D*—H···*A*	*D*—H	H···*A*	*D*···*A*	*D*—H···*A*
N1—H1···N2*A*^i^	0.86 (3)	2.30 (3)	3.077 (4)	151 (2)
N1*A*—H1*C*···N2^ii^	0.87 (3)	2.42 (3)	3.153 (4)	142 (3)
N2*A*—H2*C*···N2	0.92 (3)	2.27 (3)	3.092 (4)	149 (3)
N2*A*—H2*D*···N1*A*	0.87 (3)	2.27 (3)	2.753 (5)	115 (2)
N1*B*—H1*F*···N2*B*	0.98 (4)	2.12 (3)	2.710 (5)	117 (2)

Symmetry codes: (i) −*x*, −*y*, −*z*+1; (ii) *x*, −*y*+1/2, *z*+1/2.
